# Intentional retrieval suppression can conceal guilty knowledge in ERP memory detection tests^☆^

**DOI:** 10.1016/j.biopsycho.2013.04.012

**Published:** 2013-09

**Authors:** Zara M. Bergström, Michael C. Anderson, Marie Buda, Jon S. Simons, Alan Richardson-Klavehn

**Affiliations:** a School of Psychology, University of Kent, Canterbury CT2 7NP, UK; b MRC Cognition and Brain Sciences Unit, 15 Chaucer Road, Cambridge CB2 7EF, UK; c Behavioural and Clinical Neuroscience Institute and Department of Psychology, University of Cambridge, Downing Street, Cambridge CB2 3EB, UK; d Department of Neurology, Faculty of Medicine, Otto von Guericke University of Magdeburg, Zenit 1 Building, Leipziger Strasse 44, 39120 Magdeburg, Germany

**Keywords:** Episodic retrieval, Event-Related Potentials, Memory suppression, Guilty knowledge, Cognitive control

## Abstract

Brain-activity markers of guilty knowledge have been promoted as accurate and reliable measures for establishing criminal culpability. Tests based on these markers interpret the presence or absence of memory-related neural activity as diagnostic of whether or not incriminating information is stored in a suspect's brain. This conclusion critically relies on the untested assumption that reminders of a crime uncontrollably elicit memory-related brain activity. However, recent research indicates that, in some circumstances, humans can control whether they remember a previous experience by intentionally suppressing retrieval. We examined whether people could use retrieval suppression to conceal neural evidence of incriminating memories as indexed by Event-Related Potentials (ERPs). When people were motivated to suppress crime retrieval, their memory-related ERP effects were significantly decreased, allowing guilty individuals to evade detection. Our findings indicate that brain measures of guilty knowledge may be under criminals’ intentional control and place limits on their use in legal settings.

## Highlights

• Brain activity markers of memory retrieval are used in tests of criminal guilt.
• These tests assume neural markers of memory are outside voluntary control.
• We tested whether participants could suppress memory-related brain activity.
• Suppression was highly successful, significantly reducing guilt detection.
• Our results indicate that a core assumption of memory detection tests is unjustified.

## 1 Introduction

Recent suggestions that technological advances now allow us to decode criminal guilt from brain activity data have generated intensive interdisciplinary debate within the scientific community (Garland & Glimcher, 2006; Greely & Illes, 2007; Sip, Roepstorff, McGregor, & Frith, 2008; Wolpe, Foster, & Langleben, 2005). Several emerging companies are advertising commercial implementations of brain activity guilt detection (e.g. No Lie MRI, http://www.noliemri.com/; Brain Fingerprinting Laboratories, http://www.brainwavescience.com/), and attempts to introduce evidence from such tests in criminal trials are frequently reported in international media (Giridharadas, 2008; McCall, 2004; Miller, 2010). However, despite widespread interest and discussion, empirical data concerning the validity of these brain activity-based methods is sparse. One prominent concern is that most research to date has been conducted on compliant participants with little motivation to hide their guilt, whereas real criminals may use countermeasure strategies to avoid detection. In view of the important societal, legal and ethical implications of brain activity crime detection, it is vital to validate these methods before they are widely adopted, and, in particular, to evaluate how well they work for uncooperative suspects motivated to conceal incriminating knowledge.

Memory detection aims to establish culpability by determining from physiological or behavioural correlates of memory retrieval whether a suspect has knowledge of a crime that only a guilty person would possess (Meegan, 2008). Scalp-recorded Event-Related Potentials (ERPs) are often used in these types of test as inexpensive and non-invasive measures of real-time neural activity (e.g. Allen, Iacono, & Danielson, 1992; Rosenfeld, Angell, Johnson, & Qian, 1991; Van Hooff, Brunia, & Allen, 1996). ERPs have gained popularity as an alternative to traditional autonomic measures in memory detection studies (Lykken, 1959; see Ben-Shakhar & Elaad, 2003, for review), partly because the rapid and process-specific brain responses reflected in ERPs are believed to be more resistant to countermeasures than other physiological and behavioural measures (e.g. Lykken, 1998; see discussion in Ben-Shakhar, 2011). Recently however, researchers have challenged this assumption, showing that ERP memory detection tests may be more vulnerable than previously thought (e.g. Mertens & Allen, 2008; Rosenfeld, 2006; Rosenfeld, Soskins, Bosh, & Ryan, 2004, although see Rosenfeld et al., 2008). In this paper, we describe a countermeasure that has not been explored before in the literature, which questions one of the fundamental assumptions of brain-activity memory detection.

In an ERP version of a typical guilty knowledge test (GKT, Lykken, 1959), ERPs are recorded while participants engage in a crime-irrelevant target detection task that includes reminders of some incriminating information (e.g. Farwell & Donchin, 1991; Farwell & Smith, 2001). Participants are asked to discriminate between a set of target items (for example words presented on a computer screen) and another set of irrelevant control items (other words) by pressing one button for targets and another for irrelevants. This procedure produces an enlarged positive mid-parietal component termed the “P300” (see Polich, 2007, for review) in the ERP waveform around 300–900 ms specifically after target presentations, thought to index processes related to participants’ conscious recognition of targets as meaningful stimuli. Because P300 amplitudes are typically inversely related to the subjective probability of a stimulus (Donchin, 1981), a small proportion of targets are typically intermixed with a larger proportion of irrelevants to make targets subjectively rare, thus enhancing P300 differences. Crucially, to assess the presence or absence of guilty knowledge, a small proportion of crime reminders (“probes”) are also presented as part of the irrelevant set. To truly innocent suspects, such crime probes are indistinguishable from irrelevant items and thus elicit no special brain response. To guilty suspects, the probes stand out based on their crime-related memory status, and this recognition elicits an enhanced parietal P300 similar to targets. Thus, guilty suspects show enlarged parietal P300s to both probes and targets since both types elicit recognition, whereas innocent suspects only show enlarged parietal P300s to targets and not to probes.

Memory detection tests make the crucial assumption that reminders of incriminating information uncontrollably elicit recognition-related brain activity. This assumption gains plausibility from the fact that the GKT memory probes directly name details from the crime, thus constituting exceedingly potent retrieval cues for a personally significant event, making it appear extremely unlikely that if a related memory is present, the cue will not automatically evoke recognition and its neural markers. The inevitability of such retrieval is questioned, however, by recent evidence that the brain activity correlates of general memory retrieval may be under more voluntary control than has been previously assumed (e.g. Dzulkifli & Wilding, 2005; Herron & Rugg, 2003; Rissman, Greely, & Wagner, 2010). So far, the strongest evidence that memory retrieval can be intentionally prevented stems from the Think/No-Think (TNT) memory suppression paradigm (Anderson & Green, 2001). In this task, participants are trained on pairs of stimuli (typically weakly related words), and are later presented with the first item of each pair as a reminder, and are asked to either think of the associate item (the Think condition), or to completely prevent the associate from coming to mind by suppressing retrieval (the No-Think condition). Think and No-Think reminders are repeatedly presented, typically randomly intermixed in equal proportions. fMRI evidence from this paradigm suggests that people can engage response override mechanisms mediated by the lateral prefrontal cortex to suppress retrieval by modulating memory-related activity in the hippocampus in response to reminders (Anderson et al., 2004; Benoit & Anderson, 2012; Butler & James, 2010; Depue, Curran, & Banich, 2007; Levy & Anderson, 2012; Paz-Alonso, Ghetti, Anderson, & Bunge, 2013).

Most critically for memory guilt detection research which is predominantly ERP-based, asking participants to suppress unwanted memories in a TNT task causes memory-specific reductions of an ERP effect with similar polarity, topography and timing to the P300 component (Bergström, De Fockert, & Richardson-Klavehn, 2009a, 2009b; Bergström, Velmans, De Fockert, & Richardson-Klavehn, 2007; Hanslmayr, Leipold, Pastötter, & Bäuml, 2009; Mecklinger, Parra, & Waldhauser, 2009). However, these findings have been interpreted as voluntary suppression of the ERP marker of episodic recollection, which is specifically correlated with the amount of episodic detail that is consciously recollected in response to a reminder (e.g. Vilberg, Moosavi, & Rugg, 2006; see Rugg & Curran, 2007, for review). Because of their similar characteristics, parietal effects related to recollection and “classic” P300 effects related to stimulus evaluation are difficult to tease apart. This is particularly the case in tasks where episodic reminders are subjectively rare, such as the GKT, since recollection-related and classic P300 effects may both contribute to parietal ERP amplitudes. The episodic recollection effect is however more likely to be left-lateralized than P300 effects. Furthermore, although the P300 is highly sensitive to subjective probability, the parietal recollection effect may not be (Herron, Quale, & Rugg, 2003; see also Smith & Guster, 1993). These ERP effects are thus thought to index separable cognitive processes, although the precise relationship between them is still unclear.

The implications of the aforementioned research for criminal guilt detection tests nevertheless remain to be established, since there are many features of such tests that have not yet been explored in the context of retrieval suppression. No research has examined whether retrieval-related brain activity can be intentionally suppressed for objects or events directly named by the reminder itself, in particular when comparing these potent cues to novel control cues that are unlikely to elicit memory-related brain activity. Moreover, it remains unclear whether memory-related brain activity can be suppressed when reminders are subjectively rare, as in typical GKT research. If mnemonic control is possible under conditions of exceedingly strong and subjectively unexpected retrieval cues however, it raises the possibility that guilty suspects motivated to conceal their knowledge may be able to suppress brain activity elicited by incriminating probes during memory testing and hence elude detection.

Furthermore, prior TNT research has shown that repeatedly suppressing retrieval in response to a reminder can dramatically reduce the ubiquitous beneficial effects of reminders on retention, and even impair recall performance of the avoided memories compared to items in a baseline condition that have been neither recalled nor suppressed since initial learning (e.g. Anderson et al., 2004; Anderson & Green, 2001; Bergström et al., 2009b; Depue et al., 2007; see Anderson & Huddleston, 2011, for a review and meta-analysis). This finding implies that retrieval suppression during a guilt detection test may be successful to the extent of actually impairing later retention of the incriminating memories.

To determine whether people can control brain responses to reminders that might be expected to elicit incriminating recognition, we created a novel design that combined elements from both GKT and TNT paradigms, and asked participants to voluntarily suppress memories of a simulated crime. ERPs were recorded during three phases of a memory detection test that directly probed central details from a previous home burglary simulation. In one control phase, participants were truly innocent of the tested crime. In a second “guilty cooperative” phase, participants were asked to remember their crime. Finally, in a third “guilty uncooperative” phase, participants were asked to suppress crime recall to evade detection. Following the guilty knowledge test, we compared memory for repeatedly suppressed and repeatedly remembered crime details to memory for other details that were encountered during the initial burglary simulation but did not appear in the intervening detection phase (i.e. a baseline condition), to assess whether retrieval suppression of crime memories had lasting effects on memory accessibility.

The relative probabilities of item categories were varied across two experiments. Mirroring prior TNT research, the first experiment presented each category with equal probability, which allowed an assessment of whether retrieval-related activity to exceedingly strong reminders of a crime can be voluntarily suppressed in the absence of probability effects. The second experiment reduced the probability of probes and targets to investigate whether the neural response to crime reminders can be suppressed even when probes are subjectively unexpected. If people can suppress crime recall when motivated to do so, parietal P300^1^ amplitudes for probes should be reduced in the guilty uncooperative phase compared to the guilty cooperative phase, leading to significantly poorer guilt detection rates. If they are perfectly successful at suppressing retrieval, P300 amplitudes during suppression of guilty knowledge should be indistinguishable from those observed during the innocent control phase.

## 2 Materials and methods

### 2.1 Participants

Experiment one included data from 24 right-handed native German speakers (15 females) with a mean age of 24 (range 18–35). Experiment two included data from 24 right-handed native English speakers (14 females) with a mean age of 21 (range 18–35). All participants had no known history of neurological or psychiatric disease and had normal or corrected-to-normal vision, and gave written informed consent. Experiments were conducted in accordance with the guidelines of the local Ethics Committees of the University of Magdeburg (Experiment one) and the University of Cambridge (Experiment two).

### 2.2 Design, materials and procedure

Experiment one was conducted in German at the Otto von Guericke University of Magdeburg, Germany, whereas Experiment two was conducted in English at the University of Cambridge, UK. Visual and verbal materials were kept as similar as possible across experiments. Both experiments consisted of three main phases: (1) an initial burglary simulation; (2) a memory detection phase, where ERPs were recorded; and (3) a final test phase, where participants’ memory for the burglary task was assessed.

Upon arrival, participants completed the crime simulation task on a computer, which was designed to lead to rich, elaborative memory encoding of 24 photographs of distinct common objects (e.g. a gold watch, drawn at random for each person from a larger set of objects that were as semantically unrelated as possible) without intentional learning attempts. Participants were asked to vividly imagine being a burglar who was breaking into houses with the aim of stealing valuables. During each trial, a picture of a room interior was presented first, and participants were asked to imagine that they had broken into the room and judge whether they thought that particular room was likely to contain something valuable. Second, four numbers appeared on the background, marking particular locations in the room (e.g. a drawer). Participants were asked to search through the locations by pressing the corresponding numbers on the keyboard in order to find a hidden object. When they pressed the correct option an object appeared, superimposed on the room picture. Next, participants completed three rating tasks on the object-room picture. First, they decided whether the object was the kind of object they would expect to find in the particular room. Second, they rated its value, and third, decided whether they wanted to steal it. There was no upper time limit to respond and the computer only moved on to the next question after a response had been recorded. Each of the judgements could only be given after a 4 s delay of viewing the picture, meaning that each trial was a minimum of 16 s long.

For each participant, words naming one randomly drawn subset of objects from the burglary task (e.g. “watch”) were later presented as probes during the guilty cooperative block, and words naming another subset of objects (also randomly drawn) were presented as probes during the guilty uncooperative block. The rest of the study objects were not presented during the EEG phase, but were used as a behavioural baseline for the final memory tests. Other object words for which the pictures were not presented during study were randomly assigned to the target, irrelevant and innocent probe ERP conditions, using a different set for each phase. A prior control experiment (see next section) with an independent group that performed our simulated burglary task confirmed that they recognised these crime probes as referring to the previously encountered objects with ceiling accuracy, and overwhelmingly rated these probes as eliciting automatic, involuntary recall of the burglary objects. Thus, presenting these crime probes in our memory detection test would be expected to strongly and reflexively elicit retrieval of the associated objects stored in memory.

Following crime simulation, participants were fitted with an EEG cap and completed the memory detection phase on a computer. They were told that reminder words that referred to objects from the burglary would be presented on the screen, in order to try to make them remember details from the crime so that their brain activity could be used as criminal evidence. However, these reminder words would be intermixed with other words that did not refer to the burglary while they were performing a target detection task, which would be conducted in three blocks. Before each block of the memory detection test, participants practiced a block-specific list of target words and were instructed to detect and press one button whenever they recognised a target word, and press another button for any other words (buttons and response hand counterbalanced across participants).

Following target learning, more specific instructions for the particular memory detection block were given (order counterbalanced across participants). In the guilty cooperative block, participants were instructed that if they recognised an object word as corresponding to a picture seen during the crime simulation, they should try to remember as many details as possible about that object from the burglary (analogous to a blocked Think condition in the TNT paradigm). In the innocent control block, participants were told that they would be tested for a crime they had not committed so should simply focus on the target detection task (with the reasoning that an innocent suspect in a real criminal investigation would of course know themselves to be innocent).^2^ In the guilty uncooperative block, participants were told to try their best to completely stop any memories of the crime from coming to mind at all, without self-distraction (analogous to a blocked No-Think condition, with instructions similar to direct suppression group in Bergström et al., 2009b; Benoit & Anderson, 2012). It was strongly emphasised in all phases that they should always read and pay full attention to each word the entire time it was on the screen, and only press the “recognition” button for targets and the “non-recognition” button for any other words. Thus, there were no differences in overt response requirements across phases.

During each memory detection trial, words were presented for 2 s in white on a black background, preceded by a 1 s white fixation cross, and followed by a 0.5 s green fixation cross. Button presses and blinks were timed to the green fixation in order to avoid contaminating relevant EEG with motor-related artefacts. The proportion of items in the probe, irrelevant and target categories was manipulated across experiments. In Experiment one, each block contained an equal proportion of eight items in each category, in line with previous memory suppression ERP research that has presented items to be suppressed at equal probability to other items (e.g. Bergström et al., 2007, 2009a, 2009b; Hanslmayr et al., 2009; Mecklinger et al., 2009). This list of 24 words was presented twelve times, randomly intermixed each time. In Experiment two, each block contained four probes, sixteen irrelevants and four targets, i.e. with probabilities of approximately 0.17, 0.67 and 0.17 respectively, which are typical proportions in guilty knowledge tests (Allen et al., 1992; Farwell & Donchin, 1991; Mertens & Allen, 2008; Rosenfeld et al., 2004), also presented twelve times each randomly intermixed.

Following the guilt detection phase, participants completed two final tests that aimed to detect whether the earlier retrieval suppression manipulation affected later retention of the avoided memories. Memory was compared for repeatedly suppressed object pictures (i.e. object pictures for which corresponding words were presented as probes in the uncooperative phase) to repeatedly remembered object pictures (i.e. object pictures for which corresponding words were presented as probes in the cooperative phase) and to memory for baseline objects that were simply presented during the initial burglary simulation but did not appear in the guilty knowledge test phase. Because recognition memory for objects themselves was likely to be at ceiling (as established in our validation study for probe words), the tests focused on more difficult details of the encoding episode. All participants completed two tests in the same order: location recall and object recall. For these recall tests, participants were presented with all background room pictures together with the four marked locations where they had searched for a hidden object. First, they were asked to try to remember the location in which they had found the object during the burglary simulation task. Next they were asked to recall the object that they found in that location. There was no limit to response times in either test, and pictures stayed on the screen until an answer was given. Finally, participants completed a questionnaire where they rated their perceived difficulty and success at suppressing crime details in the uncooperative block and their perceived difficulty and success at remembering crime details in the cooperative block.

### 2.3 Validation recognition test

In order to validate that using object words as probes brought memories of the crime simulation to mind, an independent group of participants (*N* = 10) completed the crime simulation followed by an old/new recognition test, where words referring to objects from the burglary were intermixed with new object words, and participants judged whether a word corresponded to an object from the burglary. If participants responded that they recognised an object word, they next rated whether the burglary memory came to mind automatically or through intentional effort when seeing the word.

### 2.4 EEG recording and analysis

In Experiment one, EEG was recorded referenced to the left mastoid using a BrainVision BrainAmp amplifier from 60 Ag/AgCl scalp electrodes embedded in an Easycap. In Experiment two, EEG was recorded referenced to Cz using a Electrical Geodesic Netamps 200 system with a 128-channel HydroCel Geodesic Sensor Net. In both experiments, signals were acquired at 250 Hz (bandwidth 0.1–70 Hz) and analysed using EEGLAB 7 (Delorme & Makeig, 2004). The continuous EEG data were re-referenced to an average mastoid reference, filtered digitally with a band-pass of 0.3–30 Hz (two-way least-squares finite impulse response filter), and were then separated into epochs time-locked to the word stimulus onset. Concatenated epochs were corrected from artefacts using Independent Component Analysis (see Bergström et al., 2009b, for details). Any trials that still contained visible artefacts following artefact correction were removed. Next, ERPs were formed for the probe, irrelevant and target items within each guilty cooperative, guilty uncooperative and innocent block (i.e. nine ERP conditions in total) including only trials for which participants gave a correct response within the allocated time. Only a very small proportion of trials (8% in Experiment one and 6% in Experiment two) were deleted in total after these exclusion criteria.

Based on our strong a priori hypotheses, we followed a large body of previous P300 research by focusing the main analyses on the mid-parietal electrode site (Pz in 10/20 nomenclature), where P300 effects are consistently largest and where selective analyses are typically focused in GKT research. Parietal ERP amplitudes were first analysed at the group level using parametric statistics. Because memory detection tests are meant to function as diagnostic tools for determining the guilt of an individual person, we also estimated the reliability of P300 differences within each participant's single trial data using nonparametric bootstrap resampling (Efron & Tibshirani, 1986; Rosenfeld et al., 2004). Prior to bootstrap analysis, individual EEG trials were further lowpass filtered with an 8 Hz cut-off in order to increase signal-to-noise ratio. Subsequently, for each participant, a set of individual trials of the same size as the original probe set were drawn at random with replacement, and averaged to create a bootstrapped probe ERP. The same procedure was repeated using irrelevant trials, creating a bootstrapped irrelevant ERP. To create a bootstrapped “base-to-peak” measure (e.g. Rosenfeld et al., 2004), the maximum amplitude of the irrelevant P300 was measured by finding the 100 ms-long time window with the most positive amplitude within the range of 400–900 ms (this value being the difference between the baseline and the P300 peak, since the epochs were baseline-corrected prior to bootstrapping), and this value was subtracted from the comparable probe P300 maximum amplitude, creating a P300 difference value. Using 200 iterations of this procedure, a distribution of P300 difference values was created. In order to state with 95% confidence that probe P300s were more positive than irrelevant P300s, the value at 1.65 standard deviations below the mean of this distribution of differences should be greater than zero.

We compared bootstrap results for the base-to-peak P300 method above with bootstrap tests of two other statistics, mean baseline-corrected mid-parietal amplitude across the whole P300 time-window (400–900 ms) and P300 peak-to-peak difference (the difference between the maximum P300 amplitude value described above and the 100 ms-long time-window with the most negative value following the P300 peak up to a maximum latency of 1600 ms post-stimulus; Soskins, Rosenfeld, & Niendam, 2001). Classification rates across these statistics were compared at strict (95% confidence) and liberal (90% confidence) thresholds. Using these thresholds and measures, individuals for which probe P300s were reliably more positive than irrelevant P300s were identified and assigned as guilty.

In order to facilitate comparing our classification results with previous research, we also calculated Receiver-Operating Curves (ROC) and associated Area Under Curves (AUCs) from the bootstrap distributions by comparing hit-rates in the two Guilty conditions (i.e. the proportion of participants correctly identified as guilty in these conditions) with false alarm rates in the Innocent condition (i.e. proportion of participants incorrectly identified as guilty when innocent) across different thresholds. The AUC provides a useful summary statistic of classification performance irrespective of a particular threshold, allowing detection rates to be compared across studies that use different classification criteria (see Ben-Shakhar & Elaad, 2003). The AUC ranges from 0 to 1, and whereas an AUC value of 1 means perfect classification across all thresholds (people were always detected when guilty but never falsely identified as such when innocent, irrespective of the classification threshold) an AUC value of 0.5 means classification is at chance (people were no more likely to be classified as guilty in the Guilty phase than the Innocent phase). AUC values smaller than 0.5 mean that people were more likely to be wrongly classified as guilty when innocent than correctly detected when actually guilty. A previous meta-analysis found a mean AUC value of 0.87 across 42 GKT studies that used skin conductance measures and a mock-crime procedure similar to the current paradigm (Ben-Shakhar & Elaad, 2003) whereas a newer, still unpublished meta-analysis found a mean AUC of 0.93 across 32 GKT studies that used P300-based measures and a variety of protocols (Ben-Shakhar, personal communication).

In the current study, AUCs were calculated with the non-parametric trapezoid method, and the Cooperative vs. Innocent AUC was statistically compared against the Uncooperative vs. Innocent AUC by estimating the standard errors of the AUCs using jack-knife resampling and calculating a *z*-score for the difference, as recommended by Hanley and Hajian-Tilaki (1997). The *z*-score formula included a correction for correlations between AUCs induced by the within-subjects design, as estimated by the correlation between the jack-knife pseudo-values (see Hanley & Hajian-Tilaki, 1997, for details).

Finally, we also conducted a whole-head analysis in order to investigate the possibility that although guilty suspects may be successful at suppressing late positive parietal ERPs, their guilt may nevertheless be revealed by other ERP effects, such as effects related to the cognitive control processes that are recruited to suppress retrieval. Previous research has indicated that these control processes are manifest as early fronto-central or centro-parietal ERP negativities (Bergström et al., 2009a, 2009b; Mecklinger et al., 2009) or frontal slow-drift negativities (Hanslmayr et al., 2009). Because of the exploratory nature of this question, we used a data-driven approach with multivariate non-rotated spatiotemporal Partial Least Squares (PLS, McIntosh & Lobaugh, 2004). PLS is a powerful technique that allows examination of distributed patterns of spatial and temporal dependencies in the ERP data with minimal assumptions regarding the timing and distribution of potential effects. PLS analyzes the “cross-block” covariance between a matrix of dependent measures (the spatiotemporal ERP distribution) and a set of exogenous measures, in this case orthogonal contrast vectors representing differences between experimental conditions (the number of contrasts equal to the degrees of freedom), thereby constraining the solution to covariance attributable to the experimental manipulation. In nonrotated PLS (Bergström et al., 2009a, 2009b; McIntosh & Lobaugh, 2004) the sums of squares of the cross-block covariance between each contrast matrix and the spatiotemporal data matrix are directly tested for significance using random permutation test.

In the current analysis, PLS was conducted separately for each of the three detection phases, each phase split into early (0–400 ms), middle (400–800 ms) and late (800–1200 ms) time-windows in order to increase temporal resolution. Two contrasts, one testing the difference between probes and irrelevants and another testing the difference between probes and targets were assessed for significance in each time-window with 1000 permutations.

## 3 Results

### 3.1 Validation recognition test and post-test questionnaire

The recognition test confirmed that participants could discriminate between crime reminders and new words with very high accuracy (98% correct, SEM 0.7%) and very high confidence (average rating 2.9 (SEM 0.02) on a 1–3 scale where 3 is highly confident and 1 is not confident), and that crime memories came to mind very automatically (average rating 3.9 (SEM 0.06) on a 1–4 scale where 4 is automatic recall and 1 highly effortful recall). The validation test thus confirmed that crime objects were strongly encoded, and that presenting these crime probes in our memory detection test would be expected to strongly and reflexively elicit retrieval of the associated objects stored in memory. Consistent with this expectation, participants retrospectively judged uncooperative recall suppression to be more effortful (*t*(47) = 14.9, *P* < 0.0001; Cohen's *d* = 2.89, calculated as the difference between means divided by the pooled standard deviation to ensure unbiased effect size estimates; Dunlap, Cortina, Vaslow, & Burke, 1996) and less successful (*t*(47) = 11.1, *P* < 0.0001, *d* = 2.28) than cooperative crime recall, which did not interact with experiment. This finding indicates that recall in response to direct cues to crime related objects was highly automatic and had to be intentionally suppressed, and that effortful suppression was recruited in both experiments.

### 3.2 Focal ERP results at the mid-parietal site

#### 3.2.1 Group level analyses

**Fig. 1:**
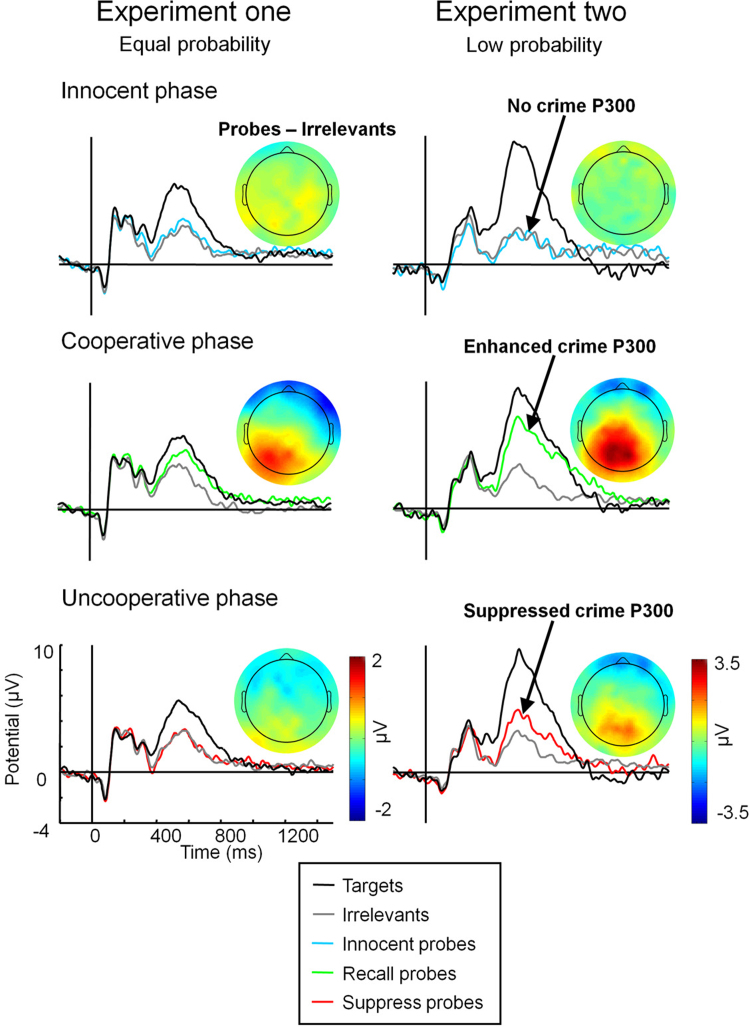
Group average mid-parietal ERPs and scalp maps contrasting different item types within blocks in Experiment one (left column) and Experiment two (right column). Topographic maps show the mean difference between probes and irrelevants between 450 and 800 ms.

In both experiments, group average ERPs at mid-parietal electrode sites showed large voluntary modulations of ERPs to crime probes depending on instructions to recall or suppress crime memories (Fig. 1), with larger probe P300s during crime recall than crime suppression. The scalp maps show that the average difference between probes and irrelevants between 450 and 800 ms in the cooperative phase displayed the canonical enhancement often found in guilty knowledge studies, relative to the innocent phase. In contrast, when subjects were uncooperative and suppressed retrieval, this enhancement was wilfully avoided. Statistics on mean ERP amplitudes between 450 and 800 ms (when the P300 effect was maximal) at the mid-parietal electrode confirmed that at the group level, voluntary modulations of memory-related P300 effects were successful, as revealed by significant interactions between the critical item categories (probe/irrelevant) and guilty phase (guilty cooperative/guilty uncooperative) in both experiments (Experiment one: *F*(1,23) = 5.57, *P* < 0.05, Partial *η*^2^ = 0.20; Experiment two: *F*(1,23) = 10.19, *P* < 0.01, Partial *η*^2^ = 0.31). These interactions between item type and guilty phase were not significantly modulated by the order in which participants completed the different phases, since adding phase order as a between subjects variable did not change the significance of the two-way interactions in either experiment, and the three-way interactions between phase order × item type × guilty phase were not significant (both *F*s < 1, *P* > 0.7).

Planned *t*-tests revealed that in Experiment one when participants were in the cooperative phase, probes elicited more positive parietal P300s than irrelevants, (*t*(23) = 3.1, *P* < 0.01, *d* = 0.48), replicating prior research with memory detection tests, although target P300s were still slightly larger than probe P300s (*t*(23) = 2.8, *P* < 0.05, *d* = 0.31). By stark contrast, when participants were asked to be uncooperative and suppress their knowledge in Experiment one, their ERPs bore remarkable resemblance to those observed in the innocent phase: probe P300s were not different from irrelevant P300s in either the uncooperative phase (*t* < 1, n.s., *d* = 0.03) in which crime knowledge was present in memory, or in the innocent phase (*t* = 1, n.s., *d* = 0.14) in which a crime memory was absent. Significantly larger P300s to targets than to probes were observed in both innocent (*t*(23) = 5.3, *P* < 0.001, *d* = 0.77) and uncooperative phases (*t*(23) = 4.1, *P* < 0.001, *d* = 0.71).

In Experiment two, where targets and probe categories were of lower probability than irrelevant items, parietal P300s to targets were indeed almost twice the magnitude of target P300s in Experiment one, consistent with previous evidence that P300 size is often inversely related to stimulus probability (Donchin, 1981). P300 amplitudes to probes were also enhanced in the low probability experiment, in particular in the cooperative phase where probe P300s were significantly more positive than irrelevant P300s (*t*(23) = 6.0, *P* < 0.001, *d* = 1.03), although target P300s were still larger than probe P300s (*t*(23) = 3.1, *P* < 0.01, *d* = 0.48). In the uncooperative phase, probe P300s were enhanced compared to irrelevant P300s (*t*(23) = 2.9, *P* < 0.01, *d* = 0.48), indicating that suppression of memory-related activity was somewhat less pronounced with low probability than with equal probability crime reminders. Similarly to Experiment one, targets were associated with significantly larger P300s than probes in both innocent (*t*(23) = 7.6, *P* < 0.001, *d* = 1.81) and uncooperative phases (*t*(23) = 6.7, *P* < 0.001, *d* = 0.99) of Experiment two.

**Fig. 2:**
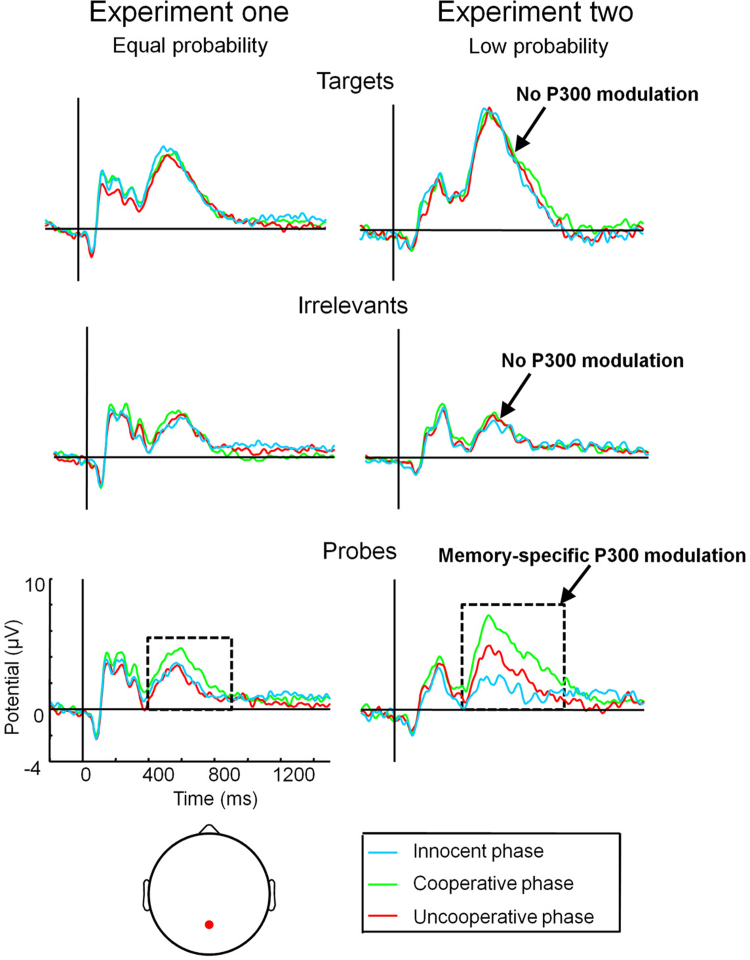
Group average mid-parietal ERPs contrasting the same item types across blocks in Experiment one (left column) and Experiment two (right column).

It is necessary to show that the reduced neural response to crime probes in Experiments one and two was not due to some phase-generic process such as paying less attention to all items during the uncooperative phase, but rather a genuine and targeted effort to control memory. Comparing ERPs across the cooperative, uncooperative, and innocent phases (Fig. 2) showed that the voluntary modulations of P300s were memory-specific because they were restricted to crime probes (dashed boxes), with no modulations of target or irrelevant P300s. *T*-tests revealed no reliable differences in P300 amplitude for targets or for irrelevants in either experiment (all *t*s ≤ 1, all *d*s < 0.17). In contrast, P300 responses to probe items were significantly reduced in the uncooperative compared to the cooperative phase in both Experiment one (*t*(23) = 2.7, *P* < 0.05, *d* = 0.50) and Experiment two (*t*(23) = 3.0, *P* < 0.01, *d* = 0.52). In Experiment one, P300 responses to probes during the uncooperative phase were reduced to the point of being indistinguishable from probes during the innocent phase (*t* < 1, n.s., *d* = 0.07); in Experiment two, uncooperative probe P300s were more positive than innocent probe P300 amplitudes (*t*(23) = 2.3, *P* < 0.05, *d* = 0.57), confirming that suppression was not complete in the low probability experiment, though clearly successful to a significant extent. These findings converge to indicate that modulations of P300 amplitude were entirely selective to crime probes, reflecting voluntary modulations of memory-specific brain activity.

#### 3.2.2 Individual guilt classification

**Fig. 3:**
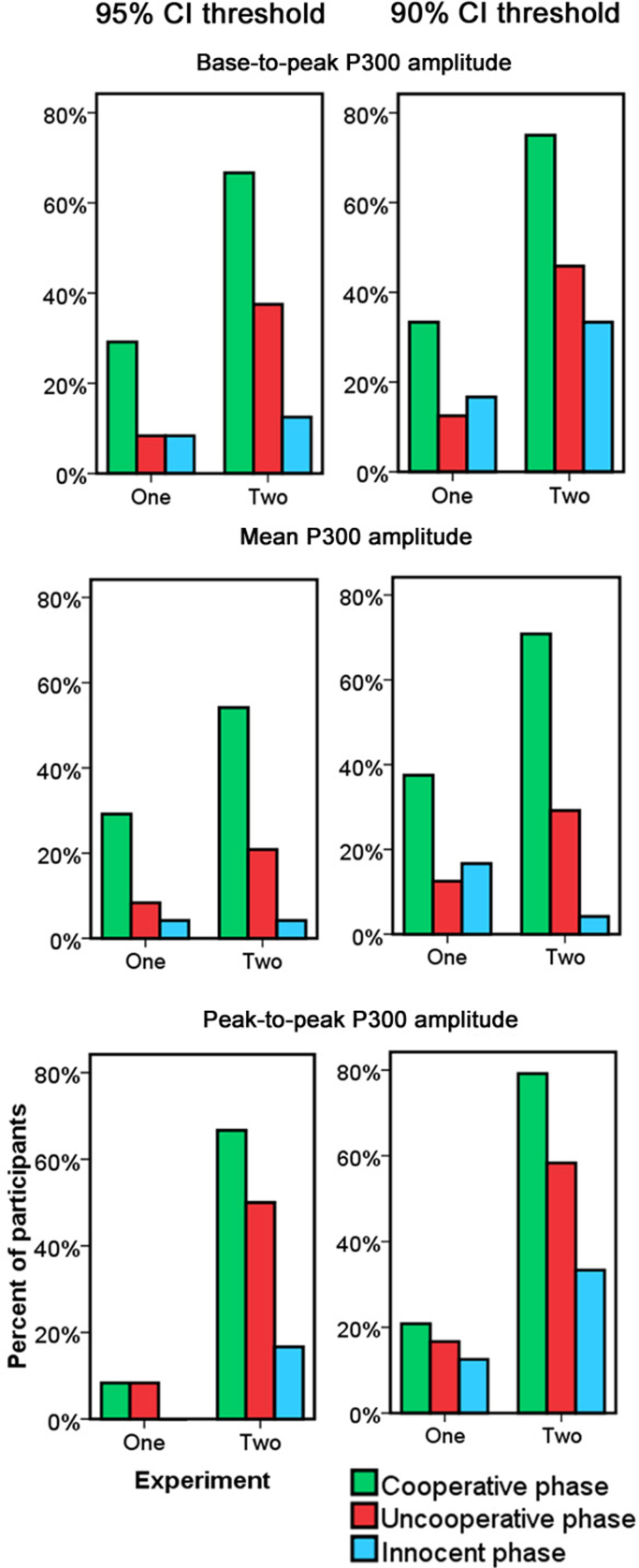
Percentage of participants classified as guilty using different statistics for the bootstrap test at different guilt classification thresholds.

The overall pattern of results in the individual guilt classification bootstrap analysis was consistent across statistics and confidence thresholds (Fig. 3). These results show that bootstrap testing of base-to-peak P300 and mean P300 amplitudes produced more accurate classification than the P300 peak-to-peak method, in particular for Experiment one. Using a strict threshold of 95% CI resulted in acceptable false positive rates for all statistics, whereas a liberal threshold of 90% CI resulted in reduced specificity (in particular for the base-to-peak and peak-to-peak P300 statistics), without much increase in sensitivity. With a liberal 90% confidence level, an unacceptably high percentage of participants were erroneously classified as guilty in the innocent phase (i.e. false alarms) for the base-to-peak and peak-to-peak P300 measures (approx. 30%). All measures showed more reasonable false alarm rates (12.5% or lower) with a strict 95% confidence threshold, except the peak-to-peak measure which was still rather high in Experiment two (17%). The peak-to-peak measure was also particularly insensitive at discriminating between probes and irrelevants across all phases in Experiment one, likely because the post-P300 negative peak (Soskins et al., 2001) was not very pronounced with equal probability probes.^3^

In Experiment one, for all measures, the percentage of individual participants classified as guilty was highly similar across the innocent phase, in which there was no crime knowledge, and the uncooperative phase, in which the participant had guilty knowledge. This similarity between innocent and uncooperative phases contrasted with the cooperative phase, in which detection rates were notably higher (with the exception of the peak-to-peak measure, as described above). In Experiment two with infrequent guilty knowledge probes, correct detection rates during the guilty phases of the experiment were overall higher than in Experiment one. Even under these more favourable conditions, however, suppressing crime memories substantially reduced detection rates compared to when participants recalled their crime.

On closer inspection, around half of participants in Experiment one did not show reliable P300 enhancements to targets compared to irrelevant items with any particular measure. The low detection rate in this experiment was thus likely a result of reduced signal-to-noise (SNR) ratio because of overall smaller P300s when probes were presented more frequently. In order to assess the pattern of detection for subjects with good SNR, we also tested classification for each measure and threshold when including only participants that showed a reliable target-irrelevant difference in that analysis (since this difference can be considered a benchmark against which the probe-irrelevant difference can be compared, e.g. Farwell & Donchin, 1991, see also Iacono, 2007), as presented in Table 1. In Experiment one, these analyses selectively enhanced detection rates during the cooperative phases by on average 24% whereas average detection rates for the uncooperative phases were only increased by 4%. In Experiment two, the majority of participants had reliable target-irrelevant differences so the conditionalised analysis only increased average detection rates by 4% in both phases. Thus, when including only high SNR participants, cooperative guilt classification was more similar across equiprobable and rare probes. Importantly for our conclusions, the conditionalised analysis confirmed that the low uncooperative detection rate in Experiment one was not due to low SNR. Rather, low SNR appeared to obscure the difference between cooperative and uncooperative phases, since this difference was enhanced when participants that failed to show reliable target-irrelevant differences were excluded.

The AUC summary measures of classification performance (Table 1) confirmed the general picture that, even though the group-level statistics were highly significant, individual classification was overall poor in Experiment one. In Experiment two, classification performance calculated on the basis of the Cooperative “hits” versus Innocent “false alarms” ROC was similar to previous skin-conductance GKT research (Ben-Shakhar & Elaad, 2003) but still lower than previous P300-based GKT research (Ben-Shakhar, personal communication). Importantly, classification performance was lower in both experiments and using all measures when calculated based on “hits” in the Uncooperative condition. Comparing the AUC measures with a jack-knife based paired *Z*-test (Hanley & Hajian-Tilaki, 1997) revealed significantly or marginally significantly higher Cooperative than Uncooperative AUC values for all bootstrap measures in both experiments, with the exception of the peak-to-peak measure in Experiment one since this measure failed to detect guilty knowledge in both phases of Experiment one (Table 1). The AUC analysis thus confirmed that the suppression-induced reduction in individual detection rates was not dependent on arbitrary classification cut-off points.

In sum, the individual bootstrap results confirmed that a sizeable proportion of participants successfully suppressed crime recall and evaded detection. Although exact success rates varied somewhat depending on the specific classification method and threshold, the global pattern was consistent, with 22% more participants classified as guilty when cooperating than when suppressing their crime memories, averaged across experiments, measures and thresholds.

### 3.3 ERP whole-head PLS analysis

The result of the PLS analysis testing pairwise contrasts in each of the detection phases and time windows are presented in Table 2. There were highly significant whole-head differences between targets and probes in all detection phases in both experiments, with the difference being maximally significant in the 400–800 ms time-window. The whole-head difference between probes and irrelevants was also highly significant during the cooperative phases, between 400 and 800 ms in Experiment one and between 400 and 1200 ms in Experiment two. However, there were no significant differences between probes and irrelevants in the innocent or uncooperative phases in either time-window and in either experiment. These whole-head PLS results thus captured the same P300 pattern as the focal parietal analysis (with the exception that the P300 difference between probes and irrelevants in Experiment two did not reach significance in the whole-head analysis), with no additional ERP indications of guilt across the global spatiotemporal data when participants were uncooperative and suppressing crime recall.

### 3.4 Final recall results

Mean accuracy on the final tests is presented in Table 3. On the four-choice location accuracy task, performance was above chance in all conditions, but there were no significant main effects or interactions. When participants were asked to recall which objects appeared in each room, there was a main effect of condition (*F*(2,92) = 11.4, *P* < 0.001, Partial *η*^2^ = 0.20), but no interaction with experiment (*F*(2,92) = 1.2, *P* > 0.3, Partial *η*^2^ = 0.03). The condition main effect was caused by significantly higher object recall accuracy (against a Bonferroni corrected *α* = 0.0167 for three family-wise post hoc tests) for repeatedly recalled objects than for repeatedly suppressed items (*t*(47) = 2.5, *P* < 0.0167, *d* = 0.37) and for baseline items (*t*(47) = 5.0, *P* < 0.001, *d* = 0.68), and trend-level higher object accuracy for suppressed than baseline items (*t*(47) = 2.0, *P* = 0.046, *d* = 0.27) collapsed across experiments.

## 4 Discussion

The current research is the first demonstration that memory-related brain activity normally elicited by reminders of incriminating knowledge can be intentionally suppressed. In line with typical guilty knowledge research, we used exceedingly strong retrieval cues as crime probes – object words as reminders of elaborately encoded object pictures – which would have greatly magnified recall prepotency compared to previously published retrieval suppression studies (Anderson et al., 2004; Bergström et al., 2007, 2009a, 2009b; Depue et al., 2007; Hanslmayr et al., 2009; Mecklinger et al., 2009). Moreover, ERPs to these powerful cues were compared with ERPs to previously unseen control items, unlikely to elicit memory-related brain activity. Despite these challenges, suppression of parietal ERP positivity was highly successful, reducing detection rates of guilty individuals.

### 4.1 Theoretical implications

Importantly, voluntary modulations of parietal ERP amplitudes were specific to crime probes, with no significant modulations of ERPs to targets or control items across phases in either experiment. This result is noteworthy because it confirms that the probe ERP effects were unlikely due to some phase-generic process, such as participants paying less attention to all items in the uncooperative phase. Rather, the ERP differences between probes across cooperative and uncooperative phases can be confidently interpreted as successful voluntary modulations of memory-specific brain activity. In fact, when we presented frequent reminders of a crime, guilty participants’ efforts to suppress retrieval rendered parietal amplitudes indistinguishable from those exhibited when they were innocent. This similarity of ERP profiles across the uncooperative and innocent phases suggests that when probes and irrelevants are equally probable, suspects can voluntarily suppress retrieval and modulate neural responses to crime probes, disguising stored knowledge so that they appear innocent. This result is thus an important extension on previous findings that the late parietal ERP positivity that is typically interpreted as a correlate of episodic recollection is largely under voluntary control (Bergström et al., 2007, 2009a, 2009b; Dzulkifli & Wilding, 2005; Hanslmayr et al., 2009; Herron & Rugg, 2003; Mecklinger et al., 2009). This ability to control retrieval may originate, in part, from the previously established capacity to intentionally modulate mnemonic activity in the medial temporal lobes and other brain areas involved in memory representation and retrieval (Anderson et al., 2004; Benoit & Anderson, 2012; Butler & James, 2010; Depue et al., 2007; Levy & Anderson, 2012).

In a second experiment, we reduced the probability of crime reminders to make them subjectively unexpected, in order to investigate whether probability-sensitive parietal ERP effects would be amenable to suppression. This issue is important because the majority of GKT applications use low-probability designs. A significant proportion of participants successfully concealed crime memories even under these more demanding circumstances, although suppression was somewhat less pronounced than with equal probability crime reminders. This finding suggests a boundary condition for voluntary control of memory-related brain activity, indicating that subjective expectedness is an important factor for suppression success.

The precise functional significance of this result is however unclear, because parietal ERP positivities in the P300 time-window are thought to index additive contributions from multiple independent sources (Johnson, 1986). In memory tasks, probability-sensitive cognitive processes related to stimulus evaluation (e.g. Sutton, Braren, Zubin, & John, 1965) and probability-insensitive cognitive processes that track episodic recollection success (e.g. Vilberg et al., 2006) may both produce ERP positivities that overlap at parietal scalp regions. It is therefore possible that varying the probe probability across experiments may have changed the relative contributions of these underlying component processes to scalp amplitudes. In Experiment one, parietal ERP positivities may have primarily been related to episodic recollection (ER). In Experiment two, the enhanced P300 amplitudes may have been the result of additive episodic recollection and probability-related ‘oddball’ (O) processes (i.e. ER + O). One speculative interpretation of our data is that these distinct processes are differentially susceptible to voluntary control mechanisms, with the episodic recollection-related ER component being more susceptible than the probability-sensitive O component. This account could explain why the relative difference between cooperative (ER+) and uncooperative (ER−) probes was similar across the two experiments even though ERP amplitudes to probes were overall enhanced in both conditions in Experiment two (O+). Alternatively, infrequent reminders may simply make it more difficult to engage the cognitive control mechanisms required to suppress retrieval due to a lack of preparation (cf. Hanslmayr et al., 2009) or practice.

In a data-driven whole-head analysis, we tested for alternative ERP effects that might have been used to diagnose guilt when the P300 was suppressed, such as effects related to the cognitive control processes recruited to suppress retrieval. Surprisingly, even though the behavioural data and self-reports indicated that retrieval suppression required a great deal of effort, this analysis failed to reveal reliable evidence of control-related ERP effects,^4^ in contrast to previous literature (Bergström et al., 2009a, 2009b; Hanslmayr et al., 2009; Mecklinger et al., 2009). One explanation for this discrepancy may be that previous research has required participants to switch on a trial-by-trial basis between suppressing and retrieving information from the same event context, whereas the current task required participants to suppress crime retrieval across an entire phase of the experiment. Such blocked retrieval suppression may involve more sustained patterns of brain activation than those observed in prior research. Sustained brain activity can be difficult to measure with ERPs, since ERPs are primarily sensitive to transient brain activity patterns that are time-locked to the onset of externally defined events.

The final recall test conducted after the guilt detection phase confirmed that the retrieval suppression manipulation affected later retention of the tested memories. Compared to recall for objects in the cooperative phase, objects repeatedly cued in the uncooperative phase showed reliably poorer memory, indicating that participants were able to limit crime recall despite repeated reminders. However, suppressed items were still somewhat strengthened compared to baseline objects that were never presented in the intermediate GKT task. This result contrasts with previous findings that repeatedly suppressing retrieval in response to a reminder can significantly impair recall performance of the avoided memories compared to baseline (e.g. Anderson & Green, 2001; Anderson et al., 2004; Bergström et al., 2009b; Depue et al., 2007). The trend level enhancement for suppressed items relative to baseline items independently confirms the subjectively very high difficulty participants reported for the recall suppression task, confirming that the mnemonic control evident in reduced parietal amplitude was the product of effortful control. Such great intrusiveness was expected, given that the cues directly referred to the objects themselves and thus likely shared many overlapping features with the to-be-suppressed memories (see Anderson & Spellman, 1995). Therefore, retrieval suppression is likely to be more difficult with the overlapping cues used in GKT compared to the non-overlapping cue-associate pairs used in other memory suppression research (e.g. Anderson & Green, 2001). Nevertheless, it is possible that under a different protocol, criminals may be able to suppress memories of their crime to the point of significant below-baseline forgetting.

### 4.2 Practical implications

A few previous studies have shown that countermeasures can degrade ERP-based memory guilt detection. Some have involved training participants on covert responses to irrelevant control items (e.g. Rosenfeld et al., 2004, 2008; Meixner & Rosenfeld, 2010), with the aim of increasing P300s to irrelevants and thus making probes and irrelevants more similar. Others have trained participants to make additional covert responses to target items in order to enhance their relative salience, thereby reducing the attentional resources available for processing of probes and irrelevants (Mertens & Allen, 2008). Importantly, no previous studies have assessed whether *memory-related brain activity in response to crime reminders* can be voluntarily and specifically controlled. Our study is thus the first to challenge the critical assumption that memory-related brain activity is automatically elicited when suspects are presented with crime reminders. The current results show that this assumption of memory detection tests is not always justified.

The generalizability of our findings is somewhat complicated by the multitude of different guilty knowledge protocols and classification techniques that have been developed by different groups (e.g. Allen et al., 1992; Farwell & Donchin, 1991; Rosenfeld et al., 1991, 2004). Although we kept our design as similar as possible to the type of protocol that has arguably been the most prevalent in the GKT literature, we did introduce some novel design elements in order to be able to manipulate retrieval suppression and investigate subsequent effects on memory (Anderson & Green, 2001). For example, we employed a very elaborate encoding phase to ensure that crime memories would be highly intrusive, and our manipulation used different instructions from practical applications of GKT research. The majority of applied GKT procedures use low probability probes, so although our first experiment was theoretically important for prior memory suppression work, the second experiment with low probability probes was most relevant to practical applications. Therefore, whilst our design emphasised internal validity, aspects of the design had reduced ecological validity. Furthermore, newly developed protocols have been demonstrated as more accurate and resistant to other types of countermeasures (Rosenfeld et al., 2008; Rosenfeld & Labkovsky, 2010). Whether or not retrieval suppression can be successfully applied in these alternative protocols is an empirical question. Further research is also required to determine whether suppression attempts can reduce guilt detection based on behavioural or ANS measures of memory (reviewed in Ben-Shakhar & Elaad, 2003).

It is also crucial that suppression countermeasures are assessed outside the laboratory. First, memories of a real crime may differ in intrusiveness from those of a crime simulation, which could affect suppression success. Second, real criminals will likely differ in their motivation to control retrieval from typical research volunteers. Since our aim was to experimentally demonstrate people's capacity to control memory-related brain activity, we manipulated whether our volunteers should retrieve or suppress crime memories. Of course, in a real crime setting, a suspect is more likely to attempt to suppress than intentionally retrieve crime memories. In contrast, volunteers participating in lab-based studies on guilty knowledge testing are likely more cooperative than real criminal suspects. Typical research volunteers without countermeasure instructions will have little motivation to suppress retrieval, and may even intentionally retrieve crime memories in response to demand characteristics (e.g. Orne, 1962). Such cooperation from volunteers would lead to an over-estimation of the test's ability to detect memories in a real crime setting.

Nevertheless, despite methodological differences, the current research has important practical implications because it challenges the assumption that memory-related brain activity is outside of voluntary control, and hence more resistant to countermeasures than other physiological and behavioural measures (e.g. Lykken, 1998; see Ben-Shakhar, 2011). Our findings showed an unprecedented degree of success at intentional control over memory-related brain activity, with little special training, and that this control can be marshalled as an effective countermeasure during memory detection tests. These results thus demonstrate that in principle, retrieval suppression poses a challenge to guilt detection tests that rely on brain-activity markers of memory.

The ability of uncooperative suspects motivated to disguise their guilt to suppress memory-related neural activity established here raises concerns regarding the validity of ERP-based memory detection tests as a means to establish criminal guilt or innocence. Together with previous evidence (Mertens & Allen, 2008; Rosenfeld et al., 2004), the current findings point to a specific problem with enhanced false negatives in memory detection tests, meaning it is particularly risky to conclude that a suspect is innocent based on a negative result. These concerns are important because it has been argued that ERP P300-based methods can reliably demonstrate that a suspect lacks knowledge of a crime, and that these techniques have been instrumental in winning the release of criminal suspects in real legal cases (see McCall, 2004). Although not every participant managed to avoid detection under all circumstances, our results indicate that, even under situations most favourable for guilt detection, at least an additional 20% of guilty criminal suspects could be misclassified and potentially set free as a result of intentional retrieval suppression, which may have dire real life consequences. Although scepticism has prevailed when this type of evidence has been considered in US legal cases, both the methods and their legal standing are still evolving, as recently noted in Science Magazine (Miller, 2010).

## 5 Conclusions

Across two experiments, we have demonstrated that the absence of a reliably enhanced brain response to crime reminders is not unequivocal evidence that relevant memories are absent in that person's brain. Instead, the presence or absence of memory-related brain activity appears to primarily track whether that person is having a subjective experience of remembering (cf. Allen & Mertens, 2009; Rissman et al., 2010). An absence of memory-related brain activity to reminders thus indicates that suspects are not remembering an associated crime *at that specific time*, but does not determine that they have no such crime memories stored in their brain. An innocent verdict in a guilty knowledge test could arise because a suspect is truly innocent, because they have forgotten the particular details of the crime that are being tested, or because they are highly motivated to disguise their knowledge and are intentionally suppressing crime memories.

## Figures and Tables

**Table 1:** Conditionalised guilt classification and Area Under Curve results across experiments and bootstrap measures.

Phase	CI	Experiment one						Experiment two					
		Base-to-peak		Mean		Peak-to-peak		Base-to-peak		Mean		Peak-to-peak	
		%	*N*	%	*N*	%	*N*	%	*N*	%	*N*	%	*N*
Conditionalised guilt classification													
Cooperative	95%	50	10	67	9	29	7	76	21	53	17	70	20
	90%	58	12	63	11	33	9	73	22	76	21	82	22
Uncooperative	95%	14	7	10	10	11	9	39	23	28	18	55	22
	90%	14	14	17	12	25	12	48	23	35	20	61	23
Innocent	95%	0	13	0	11	0	14	14	21	6	17	17	24
	90%	13	15	25	12	20	15	32	22	5	19	33	24

Areas under curves (standard errors in parentheses)													
Cooperative vs. innocent		0.60 (0.08)		0.66 (0.07)		0.47 (0.08)		0.84 (0.07)		0.87 (0.06)		0.83 (0.06)	
Uncooperative vs. innocent		0.47 (0.10)		0.52 (0.14)		0.45 (0.08)		0.69 (0.09)		0.70 (0.09)		0.76 (0.08)	

AUC difference paired *Z*-test													
*Z*-score		1.61		1.62		0.17		2.13		2.34		1.10	
*P*-value		0.054		0.053		0.431		0.017		0.010		0.077	

*Note:* CI, confidence interval; %, percentage of participants that had reliably larger P300s for probes than irrelevants out of those that also had reliable larger P300s for targets than irrelevants in the relevant analysis; *N*, number of participants included.

**Table 2:** Significance values of the contrasts in the whole-head PLS analysis as estimated by 1000 permutations.

Phase	Comparison	Experiment one			Experiment two		
		0–400 ms	400–800 ms	800–1200 ms	0–400 ms	400–800 ms	800–1200 ms
Cooperative	Probes vs. irrelevants	0.344	0.006	0.029	0.400	0.001	0.001
	Probes vs. targets	0.120	0.001	0.183	0.302	0.005	0.020

Uncooperative	Probes vs. irrelevants	0.619	0.345	0.390	0.216	0.158	0.450
	Probes vs. targets	0.023	0.000	0.236	0.042	0.000	0.009

Innocent	Probes vs. irrelevants	0.758	0.748	0.950	0.787	1.000	0.907
	Probes vs. targets	0.126	0.000	0.229	0.002	0.000	0.001

**Table 3:** Proportion correct responses across the location and object final recall tests.

Test	Condition	Experiment one		Experiment two	
		*M*	SEM	*M*	SEM
Location	Recall	0.65	0.05	0.63	0.05
	Suppress	0.55	0.04	0.67	0.04
	Baseline	0.60	0.04	0.62	0.04

Object	Recall	0.67	0.05	0.46	0.05
	Suppress	0.52	0.05	0.41	0.05
	Baseline	0.46	0.04	0.40	0.04

## Footnotes

1 We refer to the late parietal ERP positivity in these experiments as a P300 effect, in line with typical GKT research. However, as discussed, the functional significance of parietal ERP positivities in the P300 time-window is unclear, since these parietal positivities might be related both to stimulus evaluation processes and to episodic recollection.

2 One might worry that telling participants that they were innocent may have somehow led to different ERP effects in the innocent phase compared to the guilty phases. Importantly however, to preview the results, this was not the case. There were no measurable differences between target and irrelevant ERP waveforms across the three phases, demonstrating that ERPs in the innocent phase were not affected by instructions.

3 The peak-to-peak P300 measure has been proposed as superior for detecting guilty knowledge compared to other bootstrap measures (Soskins et al., 2001), and our findings appear inconsistent with this argument. However, the peak-to-peak measure relies on accurately estimating the post-P300 negative peak that was not very pronounced in our data, particularly in Experiment one. Since our experiments (particularly Experiment one) had some notable design differences from previous GKT protocols, these differences may have altered the cognitive processes involved during the task and thereby affected the ERP morphology. Thus, we do not think our results speak clearly to the issue of which bootstrap measure is generally most effective for guilty knowledge detection across different paradigms.

4 There was a trend for more negative ERPs across frontal sites for probes in the same time-window as the parietal P300, particularly in the cooperative phase (see scalp maps in Fig. 1). These frontal negativities were thus not specifically related to suppression, but rather seemed inversely related to parietal P300 positivities, and thus may reflect the negative pole of a dipolar field stemming from the same generator as the P300. Since the frontal negativities did not provide additional diagnostic information regarding guilt or innocence in our data (although see Farwell & Smith, 2001) we do not report further on these effects.
